# Age-associated declined function of endothelial progenitor cells and its correlation with plasma IL-18 or IL-23 concentrations in patients with ST-segment elevation myocardial infarction

**DOI:** 10.3389/fcvm.2024.1351567

**Published:** 2024-05-24

**Authors:** Yuanting Zhu, Guoyi Cai, Luyang Lin, Hongna Fu, Cong Zhang, Lijin Zeng, Chang Tu, Zhen Yang

**Affiliations:** ^1^ Division of Emergency Medicine, Department of Emergency Intensive Care Unit, The First Affiliated Hospital, Sun Yat-sen University, Guangzhou, China; ^2^ Department of Cardiology, The First Affiliated Hospital, Sun Yat-sen University, Guangzhou, China; ^3^ NHC Key Laboratory of Assisted Circulation and Vascular Diseases, Sun Yat-sen University, Guangzhou, China; ^4^ Guangdong Provincial Key Laboratory of Major Obstetric Diseases, Department of Gastroenterology, Guangdong Provincial Clinical Research Center for Obstetrics and Gynecology, The Third Affiliated Hospital of Guangzhou Medical University, Guangzhou, China; ^5^ Department of Cardiology, SSL Central Hospital of Dongguan City, Dongguan, China

**Keywords:** endothelial progenitor cells, aging, STEMI, IL-18, IL-23, risk scores

## Abstract

**Background:**

ST-segment elevation myocardial infarction (STEMI) persists to be prevalent in the elderly with a dismal prognosis. The capacity of endothelial progenitor cells (EPCs) is reduced with aging. Nevertheless, the influence of aging on the functionality of EPCs in STEMI is not fully understood.

**Method:**

This study enrolled 20 younger STEMI patients and 21 older STEMI patients. We assessed the Thrombolysis in Myocardial Infarction (TIMI) and Global Registry of Acute Coronary Events Risk (GRACE) scores in two groups. Then, we detected EPC migration, proliferation, adhesion, and plasma interleukin (IL)-18 and IL-23 concentrations in two groups. In addition, we analyzed the interconnection between age, EPC function, plasma IL-18 and IL-23 concentrations, and GRACE or TIMI scores in STEMI patients.

**Result:**

GRACE and TIMI scores in older STEMI patients were higher than in younger STEMI patients, whereas EPC function declined. GRACE and TIMI scores were found to have an inverse relationship with the EPC function. In older STEMI patients, plasma concentrations of IL-18 and IL-23 increased. Plasma IL-18 and IL-23 concentrations were adversely connected to EPC capacity and positively related to GRACE and TIMI scores. Moreover, age was positively correlated with plasma IL-18 or IL-23 concentrations, as well as GRACE or TIMI scores. However, age was adversely correlated with EPC function.

**Conclusion:**

In patients with STEMI, aging results in declined EPC function, which may be associated with inflammatory cytokines. The current investigation may offer new perception about mechanism and therapeutic targets of aging STEMI.

## 1 Introduction

ST-segment elevation myocardial infarction (STEMI) is a leading contributor to morbidity and death (1). STEMI in the elderly is associated with a high fatality rate, as seen by research that reported a one-year death rate of 27%, posing a severe hazard to human health (2). Currently, the global registry of acute coronary events (GRACE) risk score is employed to predict the death risk at six-months for STEMI (3), and the thrombolysis in myocardial infarction (TIMI) score can forecast the one-year mortality of STEMI (4). Reperfusion treatment is usually preferred for STEMI patients (5). Timely reperfusion treatment proportion of STEMI patients reduced with age, whereas in-hospital all-cause mortality increased (6). Therefore, exploring the underlying mechanisms of aging STEMI is of great clinical significance for its prevention and treatment.

Endothelial progenitor cells (EPCs) are involved in vascular injury repair (7). An increasing amount of research implies that aging decreases the number and functionality of EPCs, which may lead to a weakened ability to repair vascular damage (8–10). Enhanced EPC levels have been linked to a decreased risk of cardiovascular death in those with coronary artery disease (11). Nevertheless, the influence of aging on the functionality of EPCs in STEMI patients still remains unclear.

Chronic inflammation has been regarded as a primary risk factor for ageing and age-associated diseases (12). It has been reported that age was related to enhanced inflammation markers (13). Outgrowth endothelial cells (OECs) were regarded as a subtype of EPC (14). IL-8 depletion of OECs prolonged lifetime, postponed senescence, as well as augmented ability, indicating the potential association between senescence-related dysfunction and IL-8 (15). Our previous study also revealed age-associated impaired EPC function in male NSTEMI patients and the relationship between EPC function and IL-6 or IL-17 (16). These studies suggested the possible interaction between EPC function decline and interleukins in the context of aging and coronary heart disease.

Interleukin-18 (IL-18) is one of the pro-inflammatory cytokines, which has been reported to participate in cardiac inflammation and fibrosis in isoproterenol-treated mice (17, 18). IL-23 is a promoter of the cytokine IL-17, which is involved in cardiac inflammatory reactions and myocardial I/R injury (19, 20). Plasma IL-18 concentrations have been shown to be higher in healthy elderly adults than in younger adults, and plasma IL-23 concentrations were higher in centenarians than in middle-aged controls (21, 22). IL-18 can impair the function of EPCs (23). Furthermore, several studies have revealed the alteration of IL-18 and IL-23 in acute coronary syndrome (ACS) (24, 25). However, the relationship between IL-18 and IL-23 and EPC function in aging STEMI has not been studied.

Based on previous studies, we hypothesize that EPC hypofunction in aging STEMI might correlate with STEMI severity, which might be associated with plasma IL-18 and IL-23 concentrations. Therefore, we analyzed the GRACE score, TIMI score, EPC function, and plasma concentrations of IL-18 or IL-23 in younger and older patients with STEMI, as well as their interconnection. This will provide a new insight in mechanism underlying aging STEMI patients.

## 2 Method

### 2.1 Sample collection

We collected blood samples from STEMI patients, and the diagnostic criteria for STEMI were defined by elevated levels of cardiac troponin T with compatible symptom and electrocardiogram change referred to the previous study (26). Patients aged 65 years and above were categorized as the older group, while those below 65 years were categorized as the younger group. 41 STEMI patients were included in this study, comprising 20 younger patients and 21 older patients. Venous blood was acquired to measure the plasma IL-18, IL-23 concentrations as well as the circulating EPC function.

### 2.2 Estimation of GRACE and TIMI scores

According to the baseline characteristics, we calculated the GRACE score on the website (www.outcomes.org/grace) (3), and the TIMI score for STEMI patients refers to a previous study (27).

### 2.3 Evaluation of EPC function

The migrative, adhesive and proliferative function of EPCs were evaluated referring to the previous researches (8, 28–33).

EPC migration was assessed with modified Boyden chambers. After the collection of EPCs, endothelial basal medium (0.5 ml) was used to resuspend them. 2 × 10^4^ EPCs were added to the upper compartment of chamber, and the chamber was put into the 24-well culture plate, which included endothelial basal medium and VEGF with 50 ng/ml concentration. The EPCs were then incubated for 24 h in 37°C environment. Subsequently, the lower surface of the filter was rinsed and stabilized by 2% paraformaldehyde. The nuclei of EPCs were labeled with DAPI, and the migrated EPCs were quantified (8, 28, 29).

EPC proliferation was evaluated with 3-(4,5-dimethylthiazol-2-yl)-2,5-diphenyltetrazolium bromide (MTT) assay. EPCs were digested and cultivated at serum-deprived medium on 96-well culture plates with a density of 200 µl/well. Ten µl of 5 mg/ml MTT was appended after 24 h, and 4-h incubation was performed. After the removal of supernatants, 10-min incubation of EPCs with 200 µl of dimethylsulfoxide (DMSO) was performed. At 490 nm, measurements for OD were carried out (8, 28).

For evaluating the adhesion of EPCs, EPCs were gathered and mixed with basal medium containing 5% fetal bovine serum. Equal amounts of EPCs were put into dishes of culture coated by fibronectin, and 30-min incubation was performed under 37°C conditions. Then the adherent cells were quantified (30–33).

### 2.4 Measurement of plasma IL-18 and IL-23 concentrations

The plasma concentrations of IL-18 and IL-23 were detected using the ELISA method in accordance with the kit's guidelines (R&D Systems, Inc.).

### 2.5 Statistical analysis

The student *T*-test was used to identify disparities in the continuous variables of normal distribution, which were presented as mean ± SD. The Mann–Whitney *U*-test found discrepancies in the continuous variables of an abnormal distribution represented by the median (25th Percentile, 75th Percentile). Categorical variables were reported as numbers and percentages, and we applied the *χ*^2^ test or the Fisher exact test to detect differences in categorical variables. Furthermore, the relationship between continuous variables following a normal distribution was examined using Pearson correlation analysis, while Spearman correlation analysis was utilized for continuous variables displaying an abnormal distribution. The *P*-value was generated with SPSS 21.0. *P* < 0.05 was considered to be statistically significant.

## 3 Results

### 3.1 Baseline characteristics

Table 1 outlines the baseline characteristics of younger and older STEMI patients. According to Table 1, there were significant variations in age between the younger and older groups. BMI, heart rate, systolic pressure, or diastolic pressure showed no discrepancies between the younger and older groups. There were no disparities in the laboratory indicators between the two groups, including ALT, AST, UREA, Cr, LDL-C, HDL-C, TC, TG, and GLU. However, older group showed higher GRACE and TIMI scores compared to younger group.

**Table 1:** Baseline characteristics between younger and older STEMI patients.

Baseline characteristic	Younger group (*n* = 20)	Older group (*n* = 21)
Age (year)	56.00 (45.50, 59.00)	69.00 (67.00, 73.50)*
Female, *n* (%)	4 (20)	5 (24)
BMI (kg/m^2^)	25.00 ± 3.92	23.66 ± 2.84
Heart rate (bpm)	78.55 ± 7.79	79.19 ± 22.88
Systolic pressure (mmHg)	128.30 ± 14.02	126.48 ± 20.79
Diastolic pressure (mmHg)	78.20 ± 10.19	72.48 ± 12.11
ALT (U/L)	44.00 (28.50, 60.75)	38.00 (25.00, 69.00)
AST (U/L)	69.00 (34.50, 157.25)	100.00 (30.00, 185.00)
UREA (mmol/L)	4.65 (4.20, 7.05)	6.70 (4.60, 9.40)
Cr (umol/L)	74.50 (59.25, 88.50)	79.00 (69.00, 95.00)
LDL-C (mmol/L)	3.33 ± 0.80	3.12 ± 1.19
HDL-C (mmol/L)	1.03 ± 0. 21	1.12 ± 0.25
TC (mmol/L)	5.06 ± 1.09	4.87 ± 1.75
TG (mmol/L)	1.67 (1.16, 2.61)	1.36 (0.90, 1.71)
GLU (mmol/L)	10.65 (6.83, 16.13)	9.80 (8.40, 10.95)
GRACE score	136.00 (118.50, 160.00)	167.00 (155.00, 190.50)*
TIMI score	3.50 ± 1.70	5.90 ± 1.84*

BMI, body mass index; ALT, alanine aminotransferase; AST, aspartate transaminase; Cr, creatinine; HDL-C, high density lipoprotein cholesterol; LDL-C, low density lipoprotein cholesterol; TC, total cholesterol; TG, triglyceride; GLU, glucose.

* *P* < 0.05 vs. Younger group.

### 3.2 EPC function in younger and older STEMI patients

**Figure 1:**
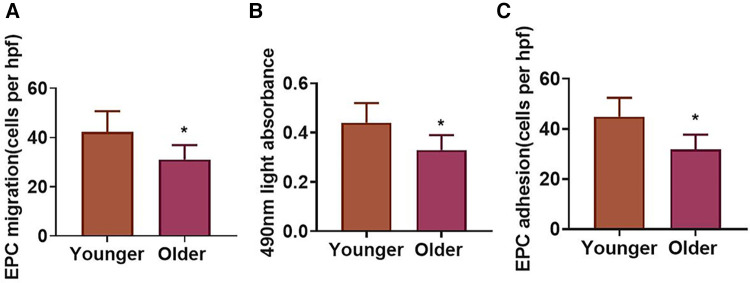
EPC function in younger and older STEMI patients. (**A–C**) Migration, proliferation, and adhesion of EPCs in younger and older groups. *P < 0.05 vs. Younger group.

In order to examine the impact of aging on endothelial progenitor cells in STEMI, we analyzed EPC capacity in younger and older STEMI patients. The migration of EPCs in the older group declined in contrast to the younger group (Figure 1A). The EPC proliferative function of the older group was also decreased as compared with the younger group (Figure 1B). The adhesive capacity of EPCs in the older group lessened in contrast to the younger group (Figure 1C).

### 3.3 The relationship between EPC function and risk scores of STEMI

**Figure 2:**
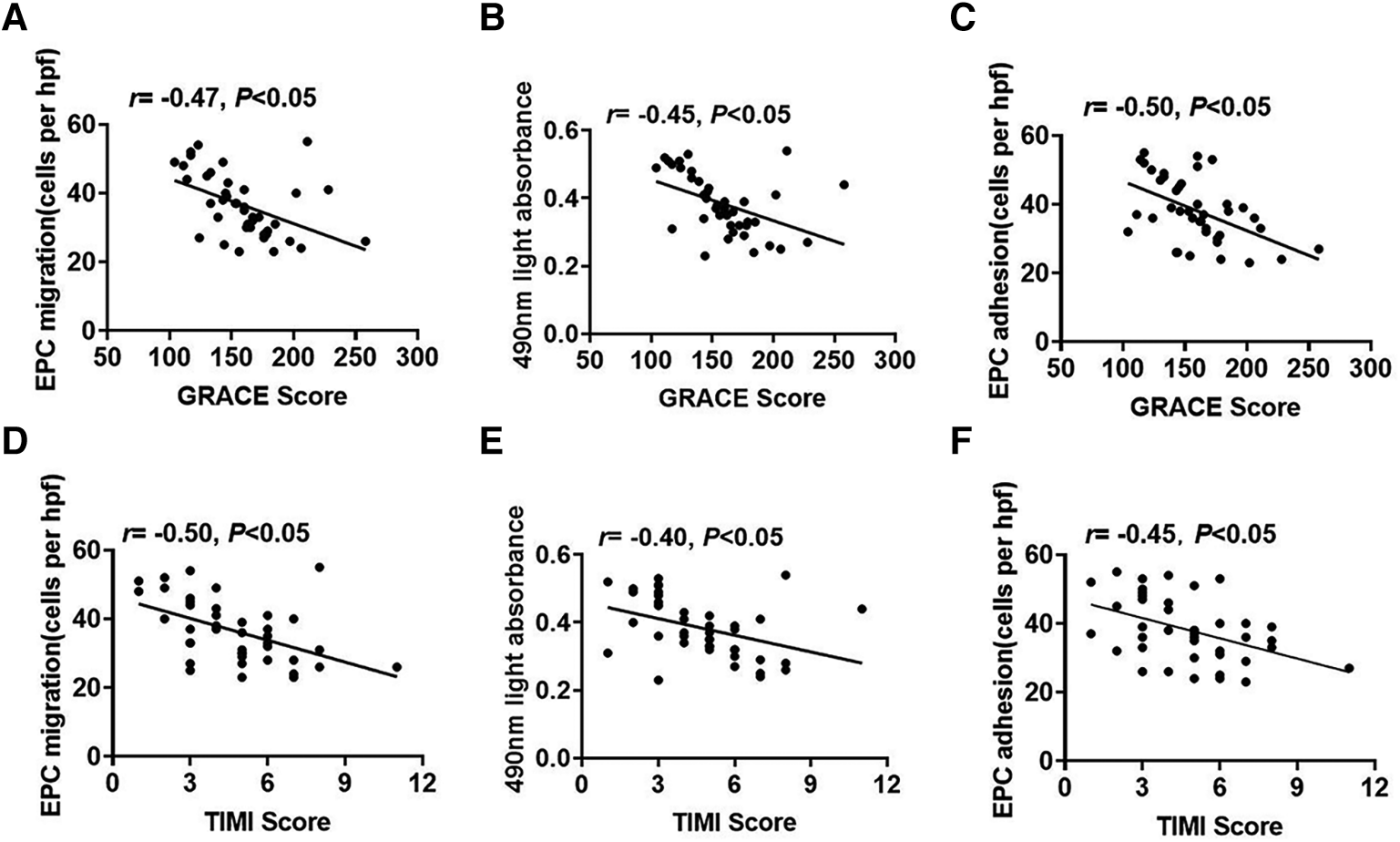
The association between EPC function and STEMI risk scores. (**A–C**) The relevance between EPC migration, proliferation or adhesion and GRACE scores. (**D–F**) The relevance between EPC migration, proliferation or adhesion and TIMI scores.

To explore the association between the EPC function and STEMI risk scores, we analyzed the correlations between the EPC function and GRACE or TIMI scores. EPC migration had negative correlations with GRACE scores (Figure 2A). Similarly, the EPC proliferative capacity was negatively related to GRACE scores (Figure 2B). The adhesive capacity was also adversely associated with GRACE scores (Figure 2C). In addition, EPC migrative capacity was negatively correlated with TIMI scores (Figure 2D). The proliferation of EPCs had an inverse relationship with TIMI scores (Figure 2E). EPC adhesion was also negatively associated with TIMI scores (Figure 2F).

### 3.4 Plasma IL-18 and IL-23 concentrations in younger and older STEMI patients

**Figure 3:**
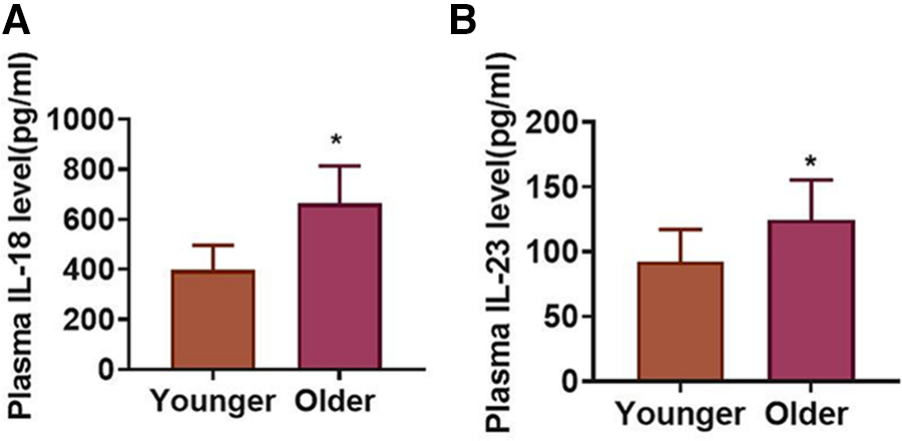
Plasma IL-18 and IL-23 concentrations in younger and older STEMI patients. (**A**) Plasma IL-18 concentrations in two groups. (**B**) Plasma IL-23 concentrations in two groups. *P < 0.05 vs. Younger group.

To study the influence of aging on inflammatory responses in STEMI, an analysis of plasma IL-18 and IL-23 concentrations was conducted in younger and older STEMI patients. Figure 3A revealed that plasma IL-18 concentrations were increased in the older group in contrast to the younger group. Meanwhile, plasma IL-23 concentrations were higher in the older group than that in the younger group (Figure 3B).

### 3.5 The association between plasma IL-18 or IL-23 concentrations and EPC function

**Figure 4:**
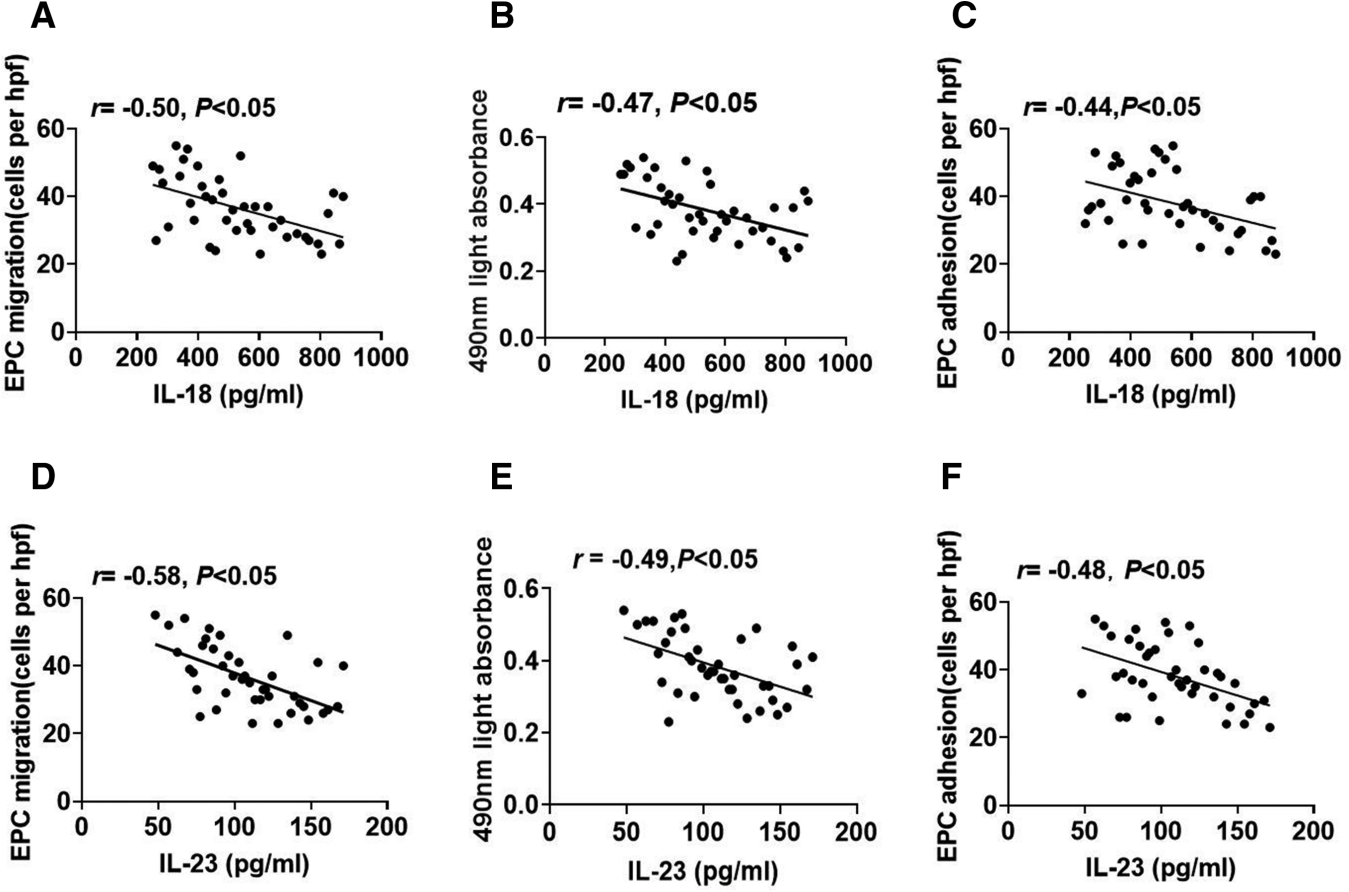
The association between plasma IL-18 or IL-23 concentrations and EPC function. (**A–C**) The relevance between plasma IL-18 concentrations and EPC migration, proliferation, and adhesion. (**D–F**) The relevance between plasma IL-23 concentrations and EPC migration, proliferation, and adhesion.

To explore the connection between inflammatory cytokines and EPC function, we analyzed the correlations between plasma IL-18 or IL-23 concentrations and EPC function. Plasma IL-18 concentrations exhibited a negative correlation with the migrative capacity of EPCs (Figure 4A). Plasma concentrations of IL-18 were also inversely correlated with EPC proliferation (Figure 4B). In addition, plasma IL-18 concentrations were negatively connected with the adhesive function of EPCs (Figure 4C). Similarly, plasma IL-23 concentrations had a negative correlation with EPC migration (Figure 4D). Figure 4E revealed the inverse association between plasma IL-23 concentrations and EPC proliferation. Plasma IL-23 concentrations were also negatively related to the adhesive capacity of EPCs (Figure 4F).

### 3.6 The correlation between plasma IL-18 or IL-23 concentrations and STEMI risk scores

**Figure 5:**
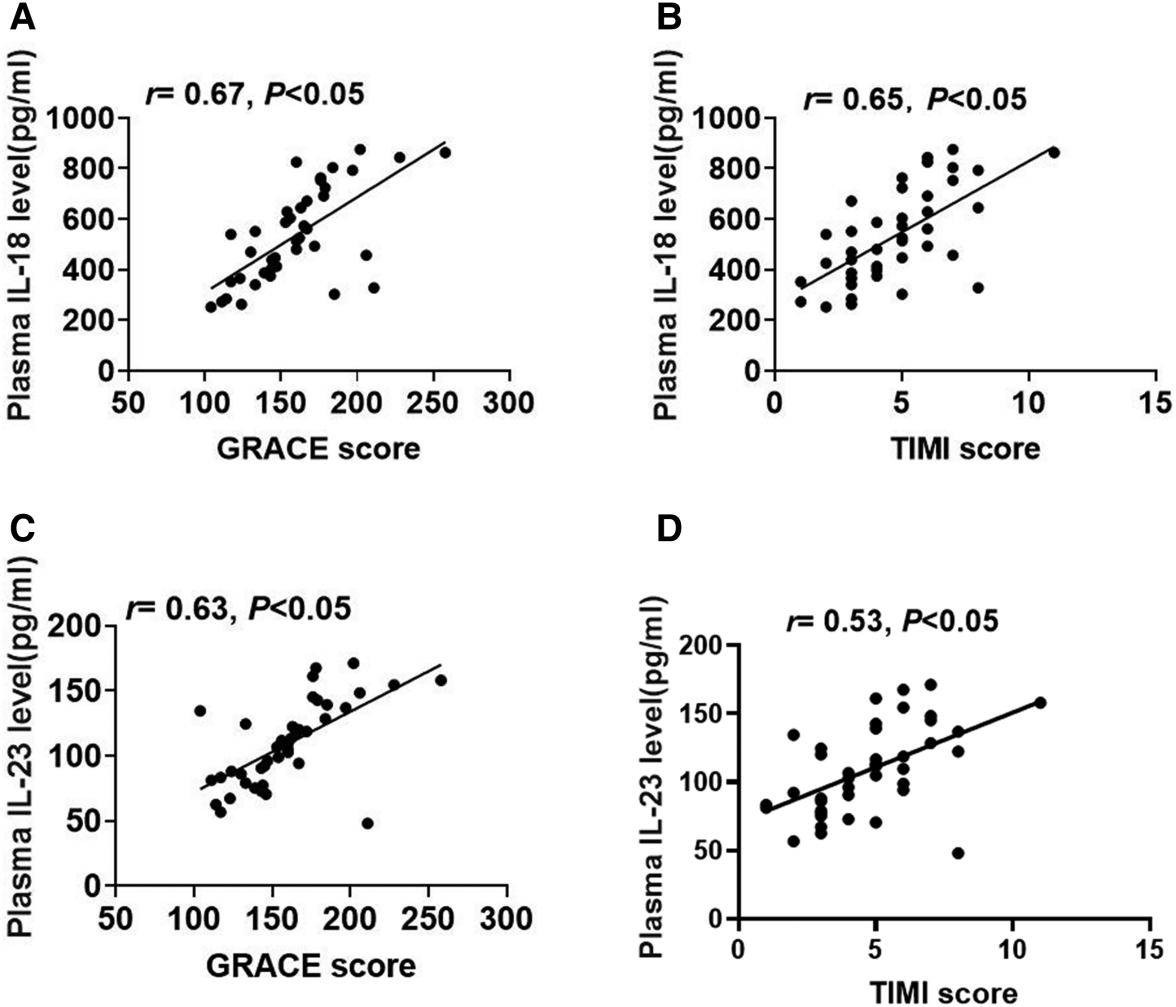
The association between plasma IL-18 or IL-23 concentrations and GRACE or TIMI scores. (**A,B**) The relationship between plasma IL-18 concentrations and the GRACE or TIMI scores. (**C,D**) The relevance between plasma IL-23 concentrations and the GRACE or TIMI scores.

To investigate the association between inflammatory cytokines and STEMI risk scores, we further analyzed the correlations between plasma IL-18 or IL-23 concentrations and STEMI risk scores. Plasma IL-18 concentrations had a positive correlation with the GRACE scores (Figure 5A). Additionally, plasma IL-18 concentrations were shown to be favorably associated with the TIMI scores (Figure 5B). Similarly, plasma IL-23 concentrations also had a positive correlation with the GRACE scores (Figure 5C). As shown in Figure 5D, plasma IL-23 concentrations were also positively related to TIMI scores.

### 3.7 The relationship between age and EPC function, plasma IL-18 or IL-23 concentrations, and risk scores of STEMI

**Figure 6:**
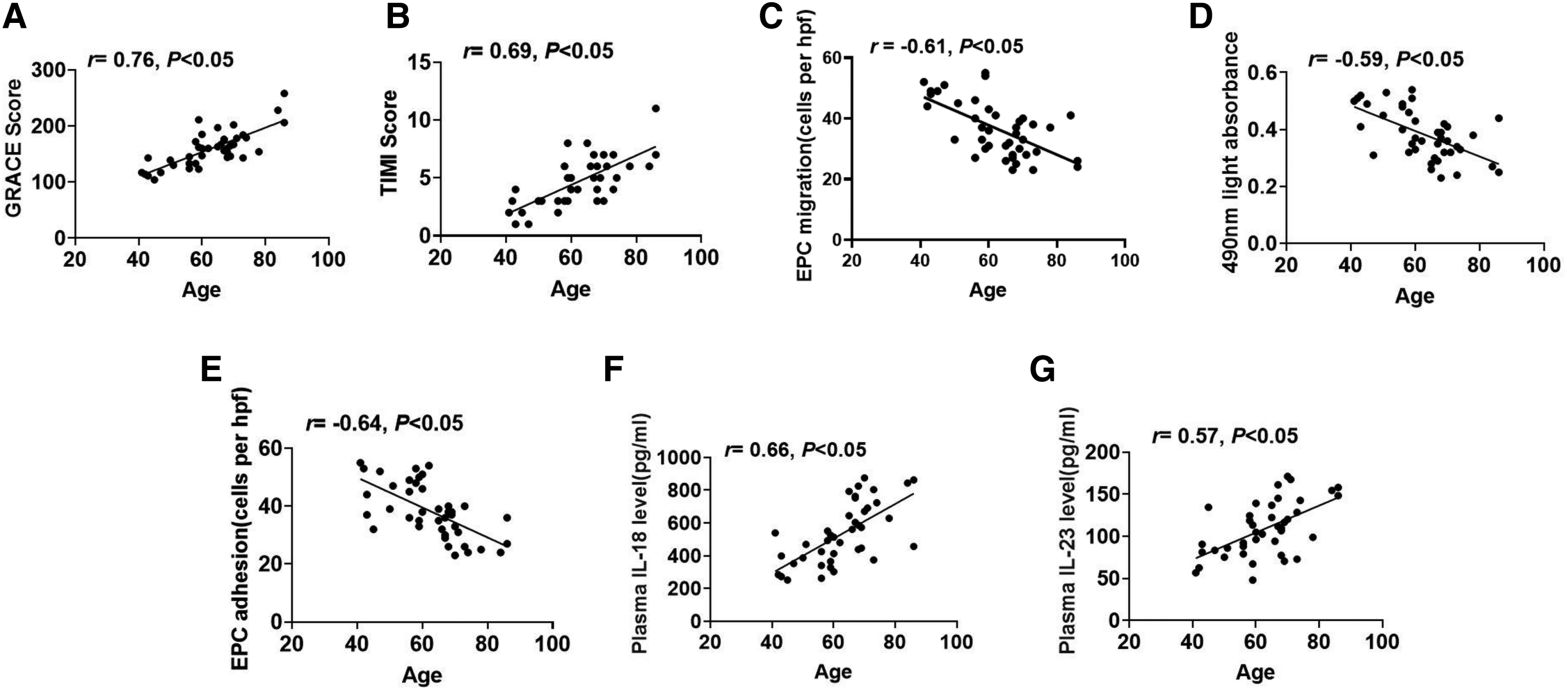
The association between age and EPC function, plasma IL-18 or IL-23 concentrations, and STEMI risk scores. (**A,B**) The relevance between age and the GRACE or TIMI scores. (**C–E**) The relevance between age and EPC migration, proliferation, or adhesion. (**F,G**) The relevance between age and plasma IL-18 or IL-23 concentrations.

As shown in Figure 6A, age exhibited a positive correlation with the GRACE scores. Similarly, age was also positively connected with TIMI scores (Figure 6B). Furthermore, age showed a negative correlation with EPC migrative capacity (Figure 6C). Analogously, age had an inverse association with proliferative function (Figure 6D). Figure 6E also indicated that age had a negative relationship with EPC adhesion. Additionally, age displayed a positive correlation with plasma IL-18 concentrations (Figure 6F). Age was also positively associated with plasma IL-23 concentrations (Figure 6G).

## 4 Discussion

Our study discovered that the older STEMI patients exhibited heightened GRACE and TIMI scores along with decreased EPC function compared to the younger STEMI patients. The EPC function was observed to be negatively correlated with the STEMI risk scores. Additionally, we observed that plasma concentrations of IL-18 and IL-23 were increased in the older STEMI patients. Plasma IL-18 or IL-23 concentrations showed an inverse correlation with EPC function and a positive correlation with GRACE and TIMI scores. Moreover, we found that age was adversely correlated with EPC function and positively correlated with IL-18 and IL-23 concentrations, as well as the GRACE and TIMI scores. These results revealed the impact of aging on EPC capacity of STEMI and its link to GRACE or TIMI scores and inflammation cytokines.

The GRACE risk score was used to predict the STEMI prognosis in the short and long terms (34). The TIMI score can also estimate short-term death risk and prognosis in STEMI patients (35). In our investigation, we found that the older STEMI patients had higher GRACE and TIMI scores, and age had a positive correlation with both GRACE and TIMI scores. Previous research also reported that age increased major adverse cardiovascular events (MACE) risk in acute MI patients, and age is considered an independent predictor of 30-day mortality in STEMI (36, 37). Taking these findings together, it is possible to conclude that age may be a significant factor for the prognosis of STEMI.

EPCs are essential for vascular injury repair and angiogenesis (7, 38). In response to vascular damage, bone marrow-derived EPCs reendothelize to repair vascular injury (39). Transplanting EPCs also increased neovascularization and inhibited myocardial fibrosis in ischemic myocardial rat (40). Suppressing oxidative stress delayed senescence and enhanced EPC functional capacity (41). Prior research discovered that colony formation ability, migration activity and tube-forming ability of EPCs in elderly AMI patients decreased compared to that of younger and middle-aged AMI patients (42). Similar to previous findings, our study further found attenuated migrative, proliferative and adhesive capacities of EPCs in older STEMI patients, as well as a negative correlation between age and EPC function. These evidences revealed the influence of aging on EPC function in patients with STEMI, indicating that aging may lead to decrement in vascular repair capacity in STEMI. In addition, we found an adverse correlation between EPC function and GRACE or TIMI scores in STEMI patients. Considering prior research that highlighted GRACE and TIMI scores as prognostic indicators of STEMI (3, 4, 34), our findings imply that age-related impairment of EPC function may be linked to the risk degree and prognosis of STEMI.

Prior research has demonstrated an age-associated increase in inflammatory cytokine levels (43). In this research, we observed that plasma concentrations of IL-18 and IL-23 also increased in older STEMI patients, and age was positively linked to IL-18 and IL-23 levels, implying that age may result in augmented inflammatory responses in STEMI. In addition, IL-18 has been shown to be expressed in the atherosclerotic plaques, and IL-18 mRNA levels were higher in unstable plaques than those in stable plaques (44). IL-23 increased responses of inflammatory and reaction of oxidative stress during the myocardial I/R process (20). Our study also discovered that plasma IL-18 and IL-23 concentrations were positively correlated with the GRACE and TIMI scores in STEMI patients, suggesting that inflammation may also be correlated with the risk level and prognosis of STEMI. Furthermore, our results revealed that in STEMI patients, plasma IL-18 and IL-23 concentrations were negatively linked with EPC function, IL-18 led to impaired function of EPCs in systemic lupus erythematosus (SLE), and circulating levels of IL-18 were associated with EPC dysfunction in SLE (23). Another investigation reported that PM (polymyositis)/DM (dermatomyositis) is related to dysregulation of EPC phenotype and function that may be partly due to abnormal IL-18 and type I IFN pathways (45). Additionally, the levels of IL-23 displayed gradual increase in patients with grade I, II, and III hypertension, and were positively related to SBP and DBP (46). It was also reported that hypertensive patients had decreased function of EPCs (47). These results and our discovery imply the possible correlation between IL-23 and endothelial repair capacity in cardiovascular diseases. Taken together with the previous studies and our findings, the elevation of inflammatory cytokine may be the mechanism underlying age-associated attenuated EPC function.

Circulating levels of IL-18 have been found to have a negative correlation with leukocyte telomere lengths (LTLs) (48), and LTL was TL proxy in other tissue (49). EPC telomere length positively correlated with their endothelial repair ability (50). Telomere attrition is known as a hallmark of aging (51), and aging can impair EPC proliferative and migrative capacity (52). Therefore, declined telomere length may be implicated in the adverse effect of IL-18 on EPC function. IL-23 was observed to elevate the expression of TNF-α under myocardial I/R injury (53). TNF-α decreased proliferative, adhesive and migrative abilities of EPCs (54). These studies imply that elevated TNF-α expression may be involved in the detrimental effect of IL-23 on EPC function. Atherosclerosis is considered to be an aging-related disease (55), and attenuated EPC proliferation and migration were observed in atherosclerotic mice (56), which hinted the potential role of EPC dysfunction in age-related diseases and aging process. Further investigations are needed to explore the underlying mechanism.

Our investigation has some significant clinical implications. First, our findings found that in older STEMI patients, there was a decline in EPC capacity and an increase in STEMI risk scores. A negative correlation has been identified between EPC capacity and STEMI risk scores. These results indicate that the capacity of EPC may be an important indicator for evaluating the risk degree and prognosis of aging STEMI. Interventions to enhance EPC capacity may be helpful in the treatment of aging STEMI. Second, older STEMI patients had higher plasma IL-18 and IL-23 concentrations in contrast to the younger STEMI patients, and the plasma concentrations of IL-18 and IL-23 were adversely associated with EPC capacity. These findings imply that age-related decreased EPC capacity in STEMI may be attributed to enhanced levels of inflammation cytokines. IL-18 inhibitor or IL-23 inhibitor (such as guselkumab or risankizumab) (57) may be the promising potential therapeutic approach for aging STEMI. Future longitudinal clinical studies remain to be performed to verify it.

There are limitations in the investigation. First, the sample size of this study is small. Future investigation with larger sample is needed to be implemented. Second, our study is a cross-sectional study. Whether EPC dysfunction predicts the outcome of aging patients with STEMI is still undiscovered. Longitudinal researches will be launched to clarify the predictive role of EPC function in the outcome of aging STEMI.

## 5 Conclusion

The present study suggested there were attenuated EPC function and increased GRACE and TIMI scores in aging STEMI. The age-related EPC dysfunction in STEMI may be related to augmented inflammation cytokines levels. The current findings may provide the novel opinions into the mechanism and treatment for aging STEMI.

## Data Availability

The raw data supporting the conclusions of this article will be made available by the authors, without undue reservation.
